# BREATHLEssness in INDIA (BREATHE-INDIA)–Study protocol for the co-design of a community breathlessness intervention in India using realist methods and intervention mapping

**DOI:** 10.1371/journal.pone.0293918

**Published:** 2023-11-02

**Authors:** Joseph Clark, Naveen Salins, Mark Pearson, Anna Spathis, David C. Currow, Siân Williams, Miriam Johnson

**Affiliations:** 1 Wolfson Palliative Care Research Centre, University of Hull, Hull, United Kingdom; 2 Department of Palliative Medicine and Supportive Care, Kasturba Medical College Manipal, Manipal Academy of Higher Education, Manipal, Karnataka, India; 3 Department of Public Health and Primary Care, University of Cambridge, United Kingdom; 4 Faculty of Science, Medicine and Health, University of Wollongong, Wollongong, Australia; 5 Joint Chief Executive Officer, International Primary Care Respiratory Group, London, United Kingdom; Bangabandhu Sheikh Mujib Medical University (BSMMU), BANGLADESH

## Abstract

**Background:**

Breathlessness that persists despite treatment of causal disease(s) is disabling, associated with high therapy-related costs and poor socioeconomic outcomes. Low resource countries bear a disproportionate burden of respiratory problems, often characterised by disabling breathlessness. Low-cost self-management breathlessness-targeted interventions are effective and deliverable in community settings but have been developed in high-income countries. We aim to understand how breathlessness self-management works in ‘real life’ populations and cultural contexts, to develop programme theory and co-design a prototype intervention to improve persistent breathlessness management in India.

**Methods and analysis:**

Using a Realist approach, Intervention Mapping and the Medical Research Council Complex Intervention Framework we will undertake two phases of work supported by our Expert Group (of respiratory, primary, palliative care physicians) and key stakeholder groups (opinion leader clinicians, community health workers and people with lived experiences of breathlessness). 1) Realist review and evaluation to identify and refine evidence and theory for breathlessness self-management, producing intervention and implementation programme theory. We will identify literature through our Expert Group, scoping searches and systematic searches (Medline, Ebscohost, CINAHL, Scopus, Psychinfo). We will map intervention components to ‘what works, for whom, and where.’ 2) Intervention development using Intervention Mapping to map intervention and implementation programme theory to intervention components, develop materials to support intervention delivery, and co-design a prototype educational intervention ready for early acceptability and delivery-feasibility testing and evaluation planning in India. Use of stakeholder groups is to allow people with experience of breathlessness and/or its management to contribute their views on content developed by our team based upon review of secondary data sources. Experts and Stakeholders are therefore not research subjects but are included as extended members of the study team and will not follow informed consent procedures. Experts and stakeholders will be acknowledge in outputs arising from our project if they wish to be. Our review conduct will be consistent with RAMESES quality standards.

**Discussion:**

At the conclusion of our study, we will have co-designed a breathlessness intervention for use in the community setting in India ready for further evaluation of: effectiveness, socioeconomic outcomes, acceptability and transferability to other low resource settings.

## Background

Breathlessness that persists despite treatment of causal disease(s) [1] causes disability and a vicious cycle of avoiding physical activity [2], deconditioning, anxiety and increasing breathlessness [3]. Those affected participate less in the workforce (absenteeism and presenteeism) [4] and face increased household expenditure on healthcare [5]. Decreased household income and increased costs risks poverty and curtailing of children’s education as they leave school in order to supplement household income or provide informal care [6]. Low-resource countries have a significant burden of respiratory illnesses, accounting for 80% of global COPD/asthma mortality [7, 8]. In India, diseases causing persistent breathlessness (e.g., chronic obstructive pulmonary disease [9], coronary heart disease [10], lung cancer [11], tuberculosis [12]) are common, mirroring smoking rates, occupational lung disease, air pollution [13] and poverty [14].

Low-cost self-management breathlessness-targeted interventions are effective and deliverable in community settings (e.g., breathing techniques, fans, lifestyle approaches, paced physical activity). These promote improved quality of life [15, 16], need little medical knowledge, and are teachable to individual patients, lay workers, family caregivers and clinical support staff. Such approaches could be scaled-up through population awareness, increasing human and social capital, and decreasing healthcare reliance [17].

Clinical practice frameworks which promote self-efficacy–the active involvement of the patient in their own care—are increasingly recognised in high-income countries and have been shown to increase patient control over their own behaviours to improve their healthcare outcomes [18]. For example, the Breathing, Thinking, Functioning model (BTF), highlights the interrelationship between breathlessness, anxiety and de-conditioning and is used to challenge cyclical unhelpful behaviours [3]. Conceptual frameworks such as “Breathing Space” highlight how when patients and clinicians actively discuss breathlessness as a distinct medical problem and not just a consequence of illness, patients more actively engage in activities which improve their resilience to breathlessness [19]. However, most evidence regarding breathlessness management has been developed in high-income settings and data from low-resource settings is largely absent.

Emerging data suggest that health beliefs may reduce intervention acceptability and need to be addressed during implementation [20]. Studies in high resource settings like the United Kingdom showed that handheld fans are effective in improving recovery time from episodes of acute breathlessness. However, patients’ and clinicians’ health beliefs about potential harms of cool airflow, particularly in warmer countries, may make fans less acceptable [21]. Conversely, as shown in a recent realist review of breathing exercises in people with COPD (in which 58% studies were from Asia, mostly from China), other components such as deep breathing, meditation and low-level exercise which make the connection between breathing and anxiety may be more acceptable in Asian cultures. However, knowledge in low-resource countries about how breathlessness interventions can improve palliative management may be patchy or non-existent, and is held back by non-inclusion in educational curricula and a lack of clinical guidelines [22].

The FRESH-AIR programme of work–explored these issues in relation to chronic respiratory disease (CRD) in diverse low-resource settings (Uganda, Kyrgyzstan, Vietnam, rural Greece and a Roma camp). This project identified a wide range of relevant beliefs and behaviours likely to influence the usefulness of an intervention targeting respiratory symptoms. For example, a common belief that chronic respiratory symptoms are caused by infection amongst community members, indicated that educational interventions were necessary ahead of introducing self-management approaches. Ayurveda is a traditional form of medicine which is widely practiced and attributes causes of illness to a disbalance of *doshas* or psycho-physiological functional principles [23]. For example, an association between by a hot-cold disbalance and illness may be particularly relevant to the acceptability of use of cold-airflow to address breathlessness exacerbations [24].

Within India, a country with a high population and cultural diversity, there is likely to be high a correspondingly complex range of beliefs and behaviours relating to the acceptability and uptake of breathlessness interventions ion. For example, yoga is increasingly used in high-income settings to support breathlessness self-management [25], but is historically associated with Hinduism and may not be acceptable to the more than 150 million people of Islam faith in India.

We will use a realist approach to explore cultural norms, context and values likely to influence acceptability and effectiveness of a breathlessness intervention through the lens of theory [26]. Using a Realist approach [27], Intervention Mapping [28] and the Medical Research Council Complex Intervention Framework [29], our objectives are, in the context of India, to:

- Understand how breathlessness self-management works in “real-life” population and individual contexts;
- Understand contexts (e.g., country, setting, community systems, beliefs, intervention components) for effective implementation;
- Develop programme theory and co-produce a prototype intervention to improve persistent breathlessness self-management in India;
- Consider adaptation for other low-resource settings, e.g., Nepal

This protocol describes preliminary work conducted by our team in order to develop a logic model and define Program Outcomes and Objectives–logic model of change (Steps 1 and 2 of Intervention Mapping) and sets out our methods to co-design a breathlessness intervention for use in the community setting in India. At the conclusion of the study, we will have developed a community breathlessness intervention and evaluation plan.

## Preliminary work

Intervention mapping is a six-step method to support systematic development of an intervention with stakeholder involvement [18]. Use of the approach has shown to result in appropriate interventions, relevant to the context in which they are delivered and increases likelihood of intervention success. Steps 1 and 2 (‘logic model of the problem and Program Outcomes and Objectives–logic model of change) have already been addressed. Drawing upon MRC guidance related to development of complex interventions, our team has developed a logic model, providing an overview of the study context and the causal assumptions we will use throughout [Table 1]. Our logic model will be used as a starting point for the further development of initial programme and intervention theories and will be developed iteratively throughout the study.

**Table 1:** Preliminary logic model.

**INTERVENTION ACTIVITIES (WITH PATIENTS)**	**INTERVENTION PROCESSES (HOW EFFECT ACHIEVED)**	**INTERMEDIATE INTERVENTION IMPACTS**	**CLINICAL AND SOCIOECONOMIC OUTCOMES**
Assess patient’s breathlessness, including impact and unhelpful/helpful beliefs; help-seeking behaviours Advocate self-management techniques (breathing techniques, fan airflow, exercise, anxiety/relaxation) Ensure each patient has a crisis plan Assess whether diagnosed/optimally managed for underlying disease	Patients understand breathlessness as a legitimate and modifiable health concern Improve patients’ knowledge, skills and self-efficacy in breathlessness management (something can be done); improve help-seeking for symptom management before crisis Address unhelpful beliefs (e.g., that exertion-related breathlessness and cool airflow is harmful) and behaviours (e.g. avoidance of physical activity; use of fan); build on helpful beliefs (breathing regulation, emotion and spirituality are linked) Ensure appropriate diagnosis and treatment of causal disease	Breathlessness is recognised as both a SIGNPOST to causal disease, and a therapeutic target in its own right Community workers actively look for and assess breathlessness Patients have access to simple, affordable self-management techniques Causal diseases are better diagnosed and managed	Increased physical and social functioning Reduced anxiety and depression Appropriate health service utilisation Increased workforce productivity Increased school attendance of children in families affected Improved quality of life
**IMPLEMENTATION ACTIVITIES**	**IMPLEMENTATION PROCESSES (HOW EFFECT ACHIEVED)**	**INTERMEDIATE IMPLEMENTATION IMPACTS**	
Training of Community Health Workers in the “Three Bs” (Breathlessness Beliefs and Behaviours management) Provision of information about breathlessness management to health and community services managers Team education and facilitation of how coherency of practice will be achieved across all team members Development and alignment of employment and supervisory structures to support delivery of “Three Bs” (breathlessness, beliefs behaviours) by Community Health Workers	Improve Community Health Workers’ knowledge, skills and self-efficacy Improved health and community services awareness of breathlessness as a modifiable problem Community health teams develop a common understanding about breathlessness management and confidently deliver together Community Health Workers are confident in their breathlessness management role, and that this is valued and supported	Community workers actively look for and assess breathlessness More patients with self-management and crisis plans More patients coming forward with breathlessness as a symptom improving rates of disease diagnosis, disease-treatment as well as breathlessness management	

**THE CONTEXT:** High prevalence of NCDs which cause breathlessness; high prevalence of severe breathlessness; low access to healthcare in many regions/areas; cultural beliefs about disease & healthcare

### Preliminary programme and intervention theory

Undertaking Steps 1 and 2 of Intervention mapping enabled us to develop our initial intervention and implementation programme theories. A programme theory aims to explain why, how and under what conditions, a set of planned actions contributes towards desired outcomes [30]. Our overarching *intervention* programme theory is that an intervention resonating with existing cultural healthy living beliefs, and working with individuals in family and community contexts, provides a stronger basis for addressing unhelpful beliefs about breathlessness management. An intervention programme theory acknowledges that what works in one context, may not work in another. For example, although use of a handheld fan is effective in reducing recovery time from acute episodes of breathlessness in the UK [31], negative perceptions of cool airflow as a cause of disease, mean that fans may not have the same outcome in India. That is, breathlessness reflects complex psycho-physiological interactions which may well be culturally-specific.

**Fig 1:**
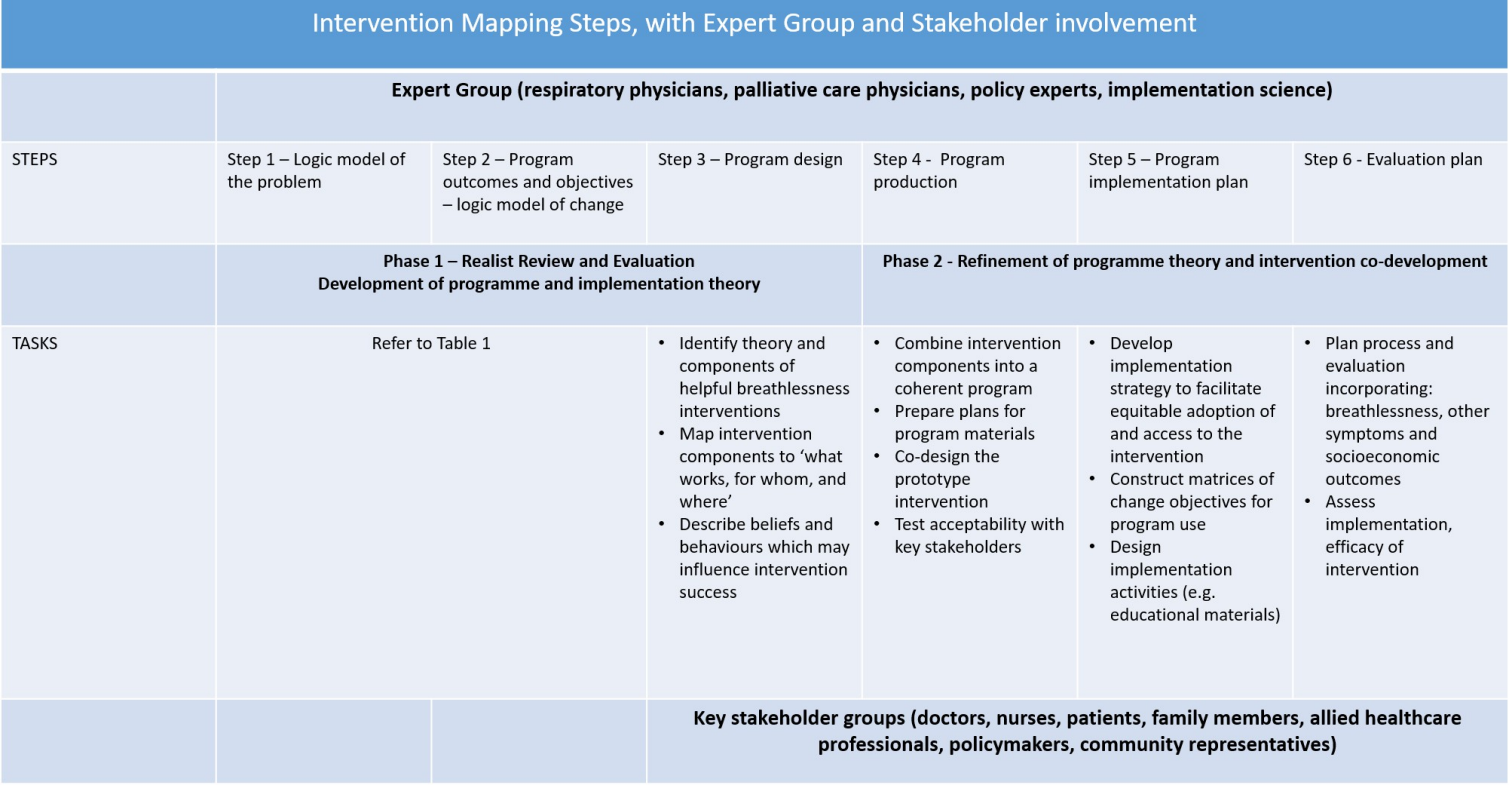
Intervention mapping framework as applied in BREATHE-INDIA.

An implementation programme theory aims to identify contextual factors likely to influence the success of implementing an intervention in a specific context. Our overarching *implementation* programme theory is that effective delivery is driven by alignment of the beliefs of a wide range of stakeholders, underpinned by low-cost to support delivery in the community. Building on the example of the handheld fan, an implementation programme theory may propose that negative beliefs about cool airflow inhibit implementation. Use of Steps 3 and 6 of Intervention Mapping, will build on our initial programme theories and causal assumptions to facilitate co-production of a breathlessness intervention, ready for evaluation. Our approach is summarised in Fig 1.

## Methods

We will undertake two phases of work. In Phase 1, we will conduct a realist review and evaluation to identify and refine evidence and theory for breathlessness self-management, producing intervention and implementation programme theory and map intervention components to ‘what works, for whom, and where’ (Step 3 of Intervention Mapping). This will be informed by our realist review’s programme theories presented in “if, then” formats to demonstrate what works, for whom, how and where. In Phase 2, we will co-design an educational breathlessness intervention using Intervention Mapping. We will map intervention and implementation programme theory to intervention components, develop materials to support intervention delivery, and co-design a prototype educational intervention ready for early acceptability and delivery-feasibility testing in India. Both phases of work will be supported by our Expert Group and five Key Stakeholder groups, comprising lay and professional people with experience of breathlessness management.

### Expert group

Project Partners form the basis of our Expert Group including experts in respiratory, primary and palliative care with links to community and other groups (e.g., Family Caregiver Education, Dream-A-Dream, Public Health Foundation India, Pallium India). Our Expert Group will meet approximately monthly via online video-technology. Further details of Expert involvement are presented alongside description of each Phase of work. They will be responsible for inviting stakeholders (Phase 1) and contributing to theory development (Phase 1 and 2).

### Stakeholder groups

We will convene ~5 stakeholder professional and lay groups from across India:

- Opinion leader physicians (primary, secondary care and public health)
- nurses/allied health professionals (primary and secondary care)
- community, education workers, lay volunteers, Accredited Social Health Activist Workers [ASHAs] primary healthcare promotors
- community/faith leaders or members
- people with experience of persistent breathlessness; family members

We will work with stakeholders as part of the realist review (Phase 1), and intervention development (Phase 2). Different Stakeholder groups will meet separately and will help to refine the future study population(s), raise breathlessness as a public health priority, and ensure future buy-in.

Our study investigators and Expert group members have wide networks across states in India. Stakeholders will be assembled purposively from rural and urban settings with variation in gender/culture/faith/language in order include a broad range of perspectives on the evidence we generate. We will develop a sampling framework, to monitor group composition and identify gaps in experiences and demographics. Once we have developed a prototype intervention, we will convene a further stakeholder group of international experts in breathlessness, community health and public health, to provide commentary on the potential for our intervention to be adapted for use in other low resource settings.

Members of the Expert Group will send a one-page study invitation, providing study information and nature of involvement to potential stakeholders. Contact details of interested stakeholders will be provided to the research team, who will make contact with the potential stakeholder, answer questions and arrange to meet.

#### Structure of meetings

Each stakeholder group will meet at least twice and be facilitated by a study team member and coordinator (interpreter available). Most will be virtual, but face-to-face (particularly community) groups can be conducted. All stakeholders will receive reimbursement for their costs involved with study participation according to local guidelines. Individuals may be interviewed if preferred. Meetings will be audio-recorded to support minute-taking, after which recordings will be deleted.

## Phases of work

### Phase 1—Realist review and evaluation: Intervention and implementation theory development

We will follow five steps to answer the research questions of our realist review [Box 1] [32]. (1) Define review scope, (2) develop initial programme theories, (3) identify evidence through systematic review, (4) study selection (5) data extraction/synthesis.

**Box 1. Research questions of realist review**

1. What are the components of interventions effective in reducing persistent breathlessness, in what settings are they effective and for which patient groups?

2. In relation to existing breathlessness interventions:

a. What mechanisms are believed to operate at different levels (individual, family, team, professional, organisational) that may explain why intended and unintended outcomes occur?

b. How do different contexts impact on the operation of these mechanisms?

3. How can knowledge of context-mechanism-outcome configurations inform the design of a community breathlessness intervention for use in India?

Using Li et al’s realist review of COPD breathing exercises [33] as our starting point, we will conduct an **expanded realist review to** explore all non-pharmacological self-management interventions for NCD-related persistent breathlessness to identify evidence and theory for self-management. Stakeholder Group meetings will be held throughout the review so that the perspectives and insights from healthcare professionals, patients and families, policymakers and community-leaders can inform the interpretation of evidence, critical reflection on emerging theories and decision-making about the focus of the review.

### Identification of evidence

Stage 1. Locate and develop existing theories about ‘how, why and where’ existing interventions are effective in reducing breathlessness. We will consult key content experts in the stakeholder group and conduct exploratory database searches to identify key literature. Our starting point will be to search for evidence developed in India, but will widen our approach to identify any evidence from other countries in South Asia as appropriate.

Stage 2. Systematic search for evidence to identify literature to refine intervention and implementation theories identified and developed in Stage 1 and identify components of effective breathlessness interventions. A search strategy will be developed drawn from keywords relevant to our initial programme theories, aiming to identify published research relevant to our review questions. We will also aim to include grey literature and policy documents highlighted as important by our expert group, or identified through other means.

We will search at least five academic databases (e.g., Medline, CINAHL, Scopus, PsychInfo, EBSCO host discovery [Indian literature]) for relevant literature. We will also conduct grey literature searches and citation searches of relevant studies. Two researchers (JC and MN) will conduct eligibility screening against inclusion/exclusion criteria. A third researcher will adjudicate disagreements to determine final inclusion of identified literature.

#### Participants/population

We will identify and include literature pertaining to the management of adults with persistent breathlessness in India.

#### Types of study to be included

Cultural beliefs and behaviours regarding breathlessness are likely to be a key mechanism for the successful development and implementation of a breathlessness intervention, although relevant information may not be reported in studies focussed on evaluation of breathlessness management interventions [34]. We will therefore adopt an inclusive approach which may include: editorials, policy documents, interventional studies, qualitative research, surveys, case reports, systematic reviews, grey literature.

#### Main outcomes

The review aims to gather evidence to inform the co-design of a breathlessness management intervention for use in India. Data collection will be structured to inform core elements of complex intervention research defined by the MRC Framework for Complex Intervention Development (considering context, developing and refining programme theory, engaging stakeholders, identifying key uncertainties, refining the intervention, and economic considerations). Key outcomes will include:

- Components of effective breathlessness interventions and mechanisms of action
- Study setting–hospital, community, hospice etc
- Study population (patient group)–lifestyle, environmental and disease-related factors causing breathlessness
- Cultural beliefs and behaviours relevant to breathlessness

#### Risk of bias and quality appraisal

We will not exclude relevant literature based on study type or quality, but will interpret evidence in context of each of these factors. To assess quality of included literature we will use a hybrid-appraisal tool used by other realist reviews to classify evidence based upon their contribution to theory, relative to the strength of evidence used for theory development. We will classify sources as conceptually-rich (rich insights grounded in theory/evidence), ‘thick‘ (rich insights, but lacking depth of conceptually-rich sources) or ‘thin‘ (of interest, but lacking explanatory detail) [35]. This tool and approach allows clear focus on stronger sources for theory identification and development whilst still allowing for the inclusion of weaker sources that can make an important, if lesser, contribution.

#### Data extraction

Data will be extracted by two researchers to an extraction form we will develop to facilitate mapping of causal links between context, mechanisms and outcome configurations.

#### Data synthesis

Data will be synthesised using realist logic [17], to build an explanatory framework of how and why mechanisms lead to certain events by analysing within and between configurations, identifying how these may differ according to context. Synthesis will be conducted through a process of reasoning. We will use principles of: juxtaposition, reconciliation, adjudication and consolidation to develop a series of questions aimed at building a multifaceted account [Table 2] [36].

**Table 2:** Analytical approach to data synthesis and theory development.

Concept	Definition	Illustrative questions arising	Theory development
Juxtaposition	Where evidence about interventions or implementation in one source enables insights into evidence about outcomes in another source	• Is this evidence/data novel? (i.e. stand-alone, not supported by confirmatory evidence)	• Does evidence identified in Step 2 confirm or challenge our intervention and implementation programme theories • What modifications are required?
Reconciliation	Where results differ in apparently similar circumstances (further investigation in the form of stakeholder consultation or iterative database searches for further evidence may be required).	• Does this account challenge the explanations made in related accounts? • Does this account add important refinements to the understanding of contexts, mechanisms, or outcomes made in related accounts?	
Adjudication	Appraisal of methodological or theoretical strengths or weaknesses.	• Is evidence drawn from studies of low methodological quality, credible/helpful for inclusion within theory development?	
Consolidation	Where evidence about mechanisms and outcomes is complementary and enables a multi-faceted explanation to be built.	• What are the preferences of stakeholders in the presence of robust, but competing explanations?	

#### Expert group and stakeholder involvement in Phase 1

Our Expert Group will meet monthly to provide expert comment on the initial and developing programme theories. In parallel, each key stakeholder group will at least twice during Phase 1 to provide expert and lay views on the relevance, credibility and usefulness of programme theories. For each meeting of all groups, a researcher and study co-ordinator will produce explanatory vignettes to present to Experts and Stakeholders, summarising emerging assumptions based upon the literature and previous meetings in a feedback loop.

At the conclusion of our realist review, we will have designed and refined our intervention and implementation theories incorporating the views and experiences of our Expert and Stakeholder groups. Barriers and facilitators for intervention success will be identified ahead of intervention development. Our review will be conducted consistent with RAMESES quality standards [37].

### Phase 2—Refinement of programme theory and intervention co-development

#### Aims

- Using findings from our realist review, we will refine our programme theories with our key stakeholders to develop a conceptual platform
- Use Intervention Mapping to identify uncertainties and inform future feasibility testing
- To develop co-design a prototype education intervention deliverable in the context of India with our key stakeholders ready for feasibility testing and evaluation of effect.

**Study design.** Parallel **co-design intervention development** will use the updated MRC Framework for Complex Intervention Development [17]. The MRC guidance divides the research process in to four phases: intervention development, feasibility, evaluation and implementation [24]. Our developmental project primarily aims to develop an intervention but will inform future work by identifying key uncertainties relevant to feasibility, implementation and evaluation. We will do so by addressing core elements relevant to each phase of complex intervention research: considering context, developing and refining programme theory, engaging stakeholders, identifying key uncertainties, refining the intervention, and economic considerations.

#### Expert group and stakeholder involvement in Phase 2

Drawing on our expertise in co-designing breathlessness interventions [38], we will involve each of our stakeholder groups in an iterative co-design process. Phase 1 will have co-developed initial, intervention and implementation programme theories. In Phase 2, we will present key components and mechanisms of action of identified breathlessness interventions, to gather views of different groups regarding how findings confirm or challenge intervention and implementation programme theories, to identify necessary refinements.

Stakeholder comprising professionals (physicians, nurses, community workers) will meet initially, to identify components of an educational intervention which may feasibly be helpful, deliverable in the community, consistent with our initial programme theories. Components of an intervention will then be presented to lay stakeholders (community leaders, patients/families) to understand their views regarding acceptability. Refinements will be identified by the study team and Expert group and this process will be repeated in a feedback loop). Each group will meet at least twice. Where divergent views are still present, our Expert group will make decisions, prioritising the views of lay stakeholders regarding acceptability.

To structure development of potentially vast implementation aspects, we will use the Consolidated Framework for Implementation Research adapted for low- and middle-income countries (CFIR-LMIC) [39] to focus on stakeholders’ perceptions about intervention scalability and sustainability; practice team norms and collective beliefs about working effectively together; need for low-cost delivery. Combined group meetings will support translation of theory and evidence into practical intervention components and a plan for implementing and sustaining the prototype community-based breathlessness education intervention ready for evaluation [27].

We will deliver the content of a flexible, co-designed, population health, community-based education intervention, relating to breathlessness-related beliefs and behaviours e.g., physical activity and cool airflow, ready for feasibility-testing and evaluation. We will have defined populations who aren’t thriving (breathlessness lens), and the population(s) for future study, agreeing a purpose for each with aims, drivers, and how to address these. Towards the end of the study, we will convene an additional Key Stakeholder Group of international experts, to provide expert commentary on the potential of the intervention we develop to be adapted for use in other low and middle-income settings. A summary of Expert Group and Key Stakeholder Involvement in Phases 1 and 2 is presented in Table 3.

**Table 3:** Summary of expert group and key stakeholder involvement in Phases 1 and 2.

Phase of project	Stakeholders	Meetings	Roles/Objectives
Phase 1 (realist review)	Expert Group	Monthly	• To identify and invite professional lay stakeholders to participate • To identify key literature and theory to inform development of intervention and implementation programme theories • To provide expert views on the relevance, credibility and usefulness of emerging programme theories • To identify barriers and facilitators
	Lay and professional groups (n = 5)	Meeting 1	• To provide views on plausibility and acceptability of programme theories • To provide views on beliefs and behaviours relevant to breathing and breathlessness • To confirm initial programme theories
		Meeting 2	
		Optional additional meeting	
Phase 2 (intervention co-design)	Expert Group	Monthly	• To provide views on key components and mechanisms of action of identified breathlessness interventions • To support Intervention Mapping and implementation design • To provide views on scalability and relevance to other low resource settings
	Lay and professional groups (n = 5)	Meeting 3	• To provide views on the feasibility and acceptability of key components and mechanisms of action of identified breathlessness interventions • To develop components of an educational breathlessness intervention for delivery in the community • To identify refinements and agree a prototype intervention
		Meeting 4	
		Optional additional meeting	

### Ethical considerations

Conducting a realist review in a low resource country with an international team has associated challenges, including: power imbalances between stakeholders (and between stakeholders and the research team) and language differences and contextual challenges [40]. To address these challenges, we will undertake the following measures. First, we will ensure that one UK and one Indian researcher are present at all Key Stakeholder group meetings conducted in English, to mediate power differentials. Where possible, Key Stakeholder group meetings will be conducted in a local language (e.g. Hindi), in such instances, we will ensure that two Hindi-speaking researchers are present. Medicine is a hierarchical profession in India, with nurses potentially deferential to the views of doctors. We will ensure that different groups are conducted separately, to ensure that all voices can be heard. Group composition will include different cultural/faith groups. Meetings may be held together with men and women, or separately on the advice of our Expert group, to account for gender role imbalance at work and in the home to facilitate women’s voices.

### Project status

BREATHE-INDIA commenced on 1^st^ October, 2022. We have developed initial programme theories based upon key literature recommended by expert co-investigators and Partners and begun systematic searches and data extraction. Ethical approval was granted on 1^st^ of February 2023. Since then we have conducted one-off stakeholder engagements at the Indian Association of Palliative Care Conference in Bangalore, India and identified key stakeholders to join regular meetings. Stakeholder group meetings will begin in March, 2023 and will continue until then end of the project in March 2024.

### Dissemination

We will follow RAMESES publication standards and aim to publish/present findings in peer-reviewed journals and conferences. We will also prepare executive summaries for lay and professional (including policy) audiences, with infographics. All Experts and Stakeholders will be invited to an online dissemination event. Short animated intervention prototypes will be produced and available on the Wolfson Palliative Care Research Centre (University of Hull), linking with other key sites (International Primary Care Respiratory Group (IPCRG) etc.) We will also publish a methodological reflection on use of realist methods for intervention development in India as a middle-income country (with huge inequity comprising low-income and high-income populations).

#### Future work

Our future aim is to implement and evaluate our educational intervention to improve the function of people with persistent breathlessness. Future prospective evaluation will include feasibility and effectiveness testing, with outcome measurement relating to health outcomes and broader socioeconomic outcomes of a breathlessness intervention (e.g. workforce participation). We aim to ensure sustainability of implementation, if effective, by developing an intervention which may challenge unhelpful, and strengthen helpful health beliefs. Additionally, our intervention is likely to be applicable to other low-resource settings; particularly to disadvantaged groups with high persistent breathlessness prevalence, e.g., older adults, greater deprivation. We will therefore also consider how this could be adapted by involving partners from outside of India (e.g. Nepal), to reflect on aspects of the intervention we develop which are specific to the context of India and what is transferable/modifiable to other settings.

## Conclusion

Persistent breathlessness is prevalent in India, causing disability and negative socioeconomic outcomes. The BREATHE-INDIA team will conduct a realist review and evaluation to develop a breathlessness educational intervention for use in the community-setting. Our intervention will be co-designed with key stakeholder groups, comprising: clinicians, people with breathlessness and their carers and community-leaders to challenge unhelpful beliefs around breathlessness, promote self-management and quality of life. At the end of our study, we will have developed a breathlessness intervention which is low cost at the point of delivery for use in the community-setting, for further evaluation and testing.
